# Life-Threatening Cardiac Manifestations of Pheochromocytoma

**DOI:** 10.1155/2010/976120

**Published:** 2010-02-10

**Authors:** Luiz R. Leite, Paula G. Macedo, Simone N. Santos, Luiz Quaglia, Cezar E. Mesas, Angelo De Paola

**Affiliations:** ^1^Department of Clinical Cardiac Electrophysiology, Centro de Estudos em Arritmia Cardíaca, SMDB Conj. 16 Lote 5 Cs A, Brasilia-DF 71680-160, Brazil; ^2^Federal University of São Paulo, Napoleão de Barros, São Paulo 04024-002, Brazil

## Abstract

Pheochromocytoma is a catecholamine-secreting tumor of the adrenal glands, usually with benign manifestations, whose typical clinical presentation includes the triad of headache, palpitations and diaphoresis. However, a wide range of signs and symptoms may be present. In the cardiovascular system, the most common signs are labile hypertension and sinus tachycardia. Systolic heart failure and ST-segment deviations mimicking myocardial infarction have also been reported, as well as QT interval prolongation and, rarely, ventricular tachycardia. We describe a challenging diagnosis of pheochromocytoma with many cardiovascular manifestations, which could have been missed due to the absence of typical symptoms.

## 1. Introduction

Pheochromocytoma is a catecholamine-secreting tumor, usually benign and localized in the adrenal gland. The classic clinical presentation is the triad of headache, palpitations, and diaphoresis. However, many cardiac symptoms are present, and the most common signs are labile hypertension and sinus tachycardia, secondary to high levels of circulating catecholamine [1]. Less frequently, the tumor can lead to myocardial dysfunction and cardiac failure, which is reversible after surgical treatment [2–4]. Some reports also describe ST-segment elevation mimicking myocardial infarction (MI) and QT interval prolongation, but only a few cases of ventricular tachycardia (VT) have been described so far [5–10]. We report a very rare case of pheochromocytoma presenting with a wide spectrum of cardiac manifestations. 

## 2. Case Report

**Figure 1:**
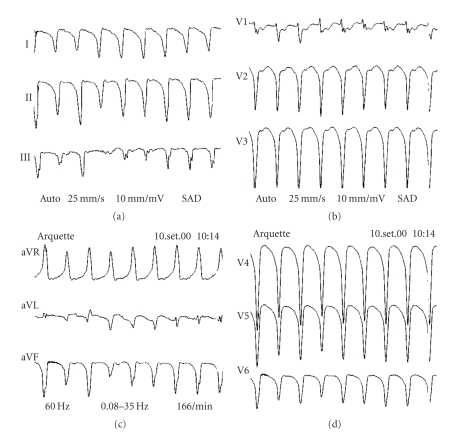
Ventricular tachycardia with left bundle branch block morphology and left axis deviation.

A 62-year-old female patient was admitted to our hospital to perform radical hysterectomy, which was complicated by postoperative incision infection. She had a history of systemic arterial hypertension, diabetes, and obesity. At the 20th day of admission, she complained of acute typical chest pain, followed by ST-segment elevation in anterolateral leads and increased CK and CK-MB serum levels, with a rise and fall typical curve [maximum of 856 mg/dL (reference range <240 mg/dL) and 154 mg/dL (reference range <24 mg/dL), resp.], and diagnosis of MI was made. She was treated with chemical thrombolysis, heparin, metoprolol, antiplatelet, and ACE inhibitor; primary angioplasty was not attempted. Twelve days after MI, she presented with palpitations and was again admitted to the ICU. She had no other symptoms, her heart rate (HR) was 160 bpm, blood pressure (BP) of 180/110 mmHg, and lung fields were clear. The ECG revealed a monomorphic VT with left bundle branch block morphology and left axis deviation (Figure 1). Ventricular tachycardia (VT) was reversed by intravenous amiodarone, and maintenance dose was initiated. In order to rule out an ischemic-related arrhythmia, a coronary angiogram was performed, showing only a 30% obstruction at the first marginal branch of the left circumflex coronary artery, and a discrete increase in the left ventricular diastolic volume. The chest X-ray was normal and the echocardiogram revealed hypokinesis of the lateral wall of the left ventricle, with a slightly decreased ejection fraction (LVEF = 56%). 

Despite amiodarone, the tachycardia episodes persisted, and procainamide was added. Further, VT episodes became refractory (4 to 5 recurrent VT per day), always well tolerated. Indeed, she presented hypertension during most episodes of VT. An electrophysiologic study was then performed aiming VT ablation, but even after many attempts including endovenous isoproterenol, the arrhythmia could not be induced. 

Sotalol was also ineffective and a trial of high doses of propranolol was used, rendering VTs less responsive to electrical cardioversion and high blood pressure during VT. Paradoxically, BP reduced after VT termination. 

**Figure 2:**
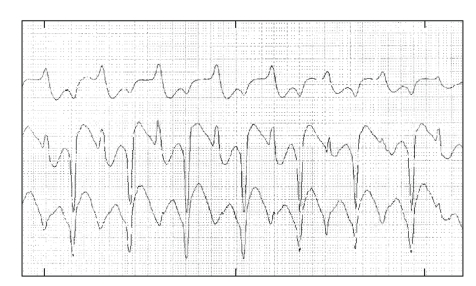
Holter monitoring revealing a bidirectional ventricular tachycardia.

**Figure 3:**
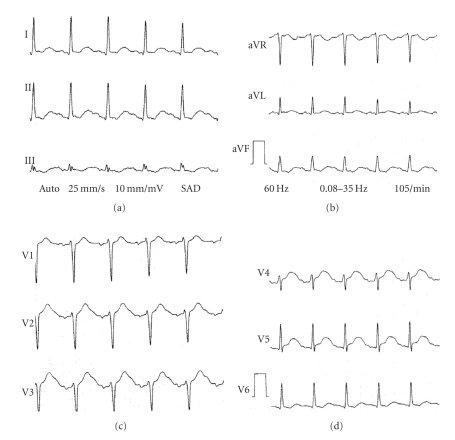
ECG in sinus rhythm demonstrating QT interval prolongation.

**Figure 4:**
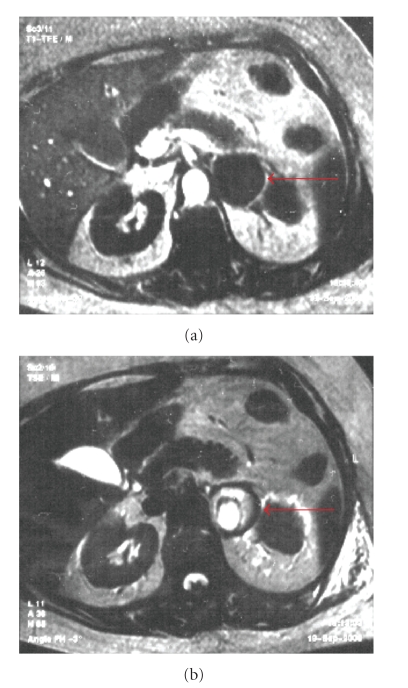
Abdominal MRI showing a large left suprarenal mass (red arrows). Right suprarenal is not seen in this view, but it was normal.

A Holter monitoring revealed bi-directional VT and an ECG showed a QTc prolongation (560 ms) (Figures 2 and 3). The characteristics of hypertension during VT and the effects of propranolol made a clinical suspicion of pheochromocytoma, which was confirmed by urinary levels of epinephrine (683 mg/24 h, reference range =<20), norepinephine (1638 mg/24 h, reference range = 15–80), dopamine (660 mg/24 h, reference range = 65–400), and vanilmandelic acid (13.9 mg/24 h, reference range =<10 mg). Magnetic resonance imaging revealed a large (46 × 48 mm) left suprarenal mass (Figure 4). No extra-adrenal pheocromocytomas was observed in the paraganglion tissue of the sympathetic nervous system or mediastinum. Differentiation with adenoma was made by in-phase and opposed phase MRI. After starting treatment with phenoxybenzamine, the BP was controlled, the QT interval normalized, and she had no recurrences of VT even after withdrawing the antiarrhythmic drugs. Unfortunately, she developed another in-hospital infection before proceeding with tumor resection. She died 2 months later as a result of multiple organ failure. Her family refused an autopsy, and histological confirmation of pheocromocytoma was not possible.

## 3. Discussion

This paper shows an uncommon presentation of a pheochromocytoma. Most of the times, this catecholamine-secreting tumor is localized at the adrenal glands and presents with the classic triad of headache, palpitations, and diaphoresis [1]. Although these classic symptoms have a high sensitivity (90.9%) and specificity (93.8%), the tumor can present in a variety of clinical pattern, which can puzzle the diagnostic investigation [1]. 

In this case, the first cardiac manifestation was MI. Some authors have already described myocardial injury mimicking MI, with or without ST-segment elevation or increased myocardial isoenzymes [3, 5–8, 11]. Our patient had a typical presentation of MI and the echocardiogram revealed segmental hypokinesis, but the coronary angiogram showed no significant obstructive lesions. It is believed that the myocardial damage is caused by ischemia secondary to increased oxygen demand and vasospasm, and a direct toxic effect of plasmatic catecholamines, but not to ischemia secondary to obstructive atherosclerosis [7]. Cardiac involvement could lead to myocardial stunning, also called acute catecholamine myocarditis, which can be viewed by a cardiac ultrasound as the “octopus sign” [8]. It can eventually evolve to cardiogenic shock requiring vasoactive drugs and left ventricular assist device implantation [4, 12]. Cardiac dysfunction is usually restored after tumor resection. This patient had only mildly compromised cardiac function though, and the “octopus sign” was not present. 

Subsequently, she presented with sustained monomorphic VT, which has already been described [2, 11, 13]. Palpitations seen in patients with pheochromocytoma are usually secondary to sinus tachycardia, but paroxysmal supraventricular and ventricular arrhythmias can also occur [2, 9, 13]. The Holter monitoring revealed bi-directional ventricular tachycardia, which is a less sensitive but a more specific finding, with only two reports of this association published [2, 14]. 

QT prolongation has been described in 16 to 35% of patients [11, 15], usually without a clear relationship with clinical events, as we observed in this case. Paulin et al. [9] have recently published a report of a patient with pheochromocytoma, monomorphic VT, and a QTc interval of 620 ms, which also decreased after treatment. In our case, although the tumor could not be surgically removed, the arrhythmia ceased and the QT interval normalized after treatment with phenoxybenzamine. 

The refractoriness of VT to standard antiarrhythmic drugs and the paradoxical increase in BP during the episodes were important clinical clues to the diagnosis. Hypertension is the most common cardiac manifestation in pheochromocytoma and is labile in up to 48% of patients. The labile nature of hypertension, a feature present in up to 48% of patients with pheochromocytoma [1], the recent MI without significant coronary arteries disease, bi-directional VT and QT prolongation also pointed to the correct underling cause. Finally, the paradoxical response to propranolol, which worsened the arrhythmic and BP control, led to the investigation of a cathecolamine-secreting tumor. Sibal et al. [16] described a case series in which patients with unsuspected pheochromocytoma had hemodynamic deterioration after beta blockage, giving rise to cardiac arrhythmia and labile BP. 

Although suprarenal mass may be incidental during MRI studies and histological confirmation of pheocromocytoma was not possible in our case, there are 3 important factors for the correct diagnosis of pheocromocytoma: the clinical presentation (especially hypertension during VT episodes), differentiation with adenoma by in-phased and opposed-phase MRI, and a good response to treatment with phenoxybenzamine, with normalization of QT interval, control of BP and VT. 

This is a good example of how challenging the diagnosis of pheochromocytoma can be, especially when it lacks the typical clinical picture, and is manifested by confounding cardiovascular system events.
